# An observational case study of hospital associated infections in a critical care unit in Astana, Kazakhstan

**DOI:** 10.1186/s13756-018-0350-0

**Published:** 2018-04-25

**Authors:** Dmitriy Viderman, Yekaterina Khamzina, Zhannur Kaligozhin, Makhira Khudaibergenova, Agzam Zhumadilov, Byron Crape, Azliyati Azizan

**Affiliations:** 1 Nazarbayev University School of Medicine (NUSOM), 5/1 Kerey and Zhanibek Khans Street, Astana, Kazakhstan 010000; 2 National Research Center for Oncology and Transplantation, Astana, Kazakhstan; 3 National Research Neurosurgery Center, Astana, Kazakhstan

**Keywords:** Intensive care unit (ICU), Hospital associated infections (HAI), Surgical site infections (SSI), Ventilator associated pneumonia (VAP), Blood stream infections (BSI), Urinary tract infections (UTI)

## Abstract

**Background:**

Hospital Associated infections (HAI) are very common in Intensive Care Units (ICU) and are usually associated with use of invasive devices in the patients. This study was conducted to determine the prevalence and etiological agents of HAI in a Surgical ICU in Kazakhstan, and to assess the impact of these infections on ICU stay and mortality.

**Objective:**

To assess the rate of device-associated infections and causative HAI etiological agents in an ICU at the National Research Center for Oncology and Transplantation (NRCOT) in Astana, Kazakhstan.

**Methods:**

This retrospective, observational study was conducted in a 12-bed ICU at the NRCOT, Astana, Kazakhstan. We enrolled all patients who were admitted to the ICU from January, 2014 through November 2015, aged 18 to 90 years of age who developed an HAI.

**Results:**

The most common type of HAI was surgical site infection (SSI), followed by ventilator-associated pneumonia (VAP), catheter-related blood stream infection (BSI) and catheter-associated urinary tract infection (UTI). The most common HAI was SSI with *Pseudomonas aeruginosa* as the most common etiological agent. The second most common HAI was VAP also with *P. aeruginosa* followed by BSI which was also associated with *P. aeruginosa* (in 2014) and *Enterococcus faecalis*, and *Klebsiella pneumoniae* (in 2015) as the most common etiological agents causing these infections.

**Conclusion:**

We found that HAI among our study population were predominantly caused by gram-negative pathogens, including *P. aeruginosa*, *K. pneumoniae*, and *E. coli.* To our knowledge, this is the only study that describes ICU-related HAI situation from a country within the Central Asian region. Many developing countries such as Kazakhstan lack surveillance systems which could effectively decrease incidence of HAIs and healthcare costs for their treatment. The epidemiological data on HAI in Kazakhstan currently is underrepresented and poorly reported in the literature. Based on this and previous studies, we propose that the most important interventions to prevent HAI at the NRCOT and similar Healthcare Institutions in Kazakhstan are active surveillance, regular infection control audits, rational and effective antibacterial therapy, and general hygiene measures.

## Background

HAI influence the quality of health care and are a major source of adverse outcomes during health care delivery [1, 2]. HAI greatly increase morbidity and mortality of patients and healthcare costs [3]. The burden of HAI in developing countries is significant, whereby the incidence can be up to 15% of total hospitalized patients, and up to 50% among ICU patients [4]. HAI are challenging to treat because the etiological agents frequently develop multidrug, extensively drug and pandrug-resistance [5]. HAI have a big economic impact on healthcare by extending ICU stay, hospital stay, and increasing the need for invasive procedures. The most common HAI are primary bloodstream infections (BSI), ventilator-associated pneumonia (VAP), urinary tract infections (UTI) and surgical site infections (SSI), with SSI being the most prevalent in some studies [2, 6]. Prevention programs for HAI which could result in positive cost-benefit ratios typically originate with laboratory data from the clinical microbiology laboratory; this provides information regarding the causative pathogenic organisms causing the HAI [6].

The incidence of HAI in ICUs is about 2 to 5 times higher than those in general inpatient departments due to many associated risk factors [1, 7]. Furthermore, antimicrobial resistance rates in ICU are much greater than in general departments [7]. In order to reduce incidence of HAI, surveillance analysis is an essential step to identify problems and implement interventions [8]. National surveillance data form the basis for prevention and control of HAI in developed countries such as the USA and Australia, but this is rarely available in many developing countries, including Kazakhstan [9]. A recent systematic meta-analysis of the burden of HAI in Southeast Asia found that the most common HAI pathogens were mostly gram-negative bacilli, and were predominantly *Pseudomonas aeruginosa*, *Klebsiella* species and *Acinetobacter baumannii*; these findings are similar to those reported for many other developing countries [9]. According to the National Healthcare Safety Network (NHSN) report in 2013, ICU-related central-line associated BSI continued to decrease, whereas urinary catheter-associated tract infection rates increased in the majority of ICU types [10]. Several studies conducted in developing countries similar to ours, reported their findings in the literature; one study from Kuwait reported that their VAP rate was 4.0 per 1000 mechanical ventilator days, the central line–associated BSI rate was 3.5 per 1000 central line days, and the catheter-associated urinary tract infection (CAUTI) rate was 3.3 per 1000 urinary catheter days [11]. Another study from Ecuador showed that device associated HAIs rates in their ICUs were higher than the United States CDC/NSHN rates and similar to International Nosocomial Infection Control Consortium (INICC) international rates [12].

In an effort to evaluate the local HAI situation in Kazakhstan, we conducted an observational case study to assess incidence of the different types of HAI over a period of two years in 2014 and 2015. Our goal was to investigate if the patterns regarding type of HAI cases and the etiological agents were consistent from year to year, therefore we performed an analysis and comparison of data from at least two years. The data from the study was retrospectively analyzed in our effort to identify the causative HAI bacterial pathogens. We aimed to assemble and analyze epidemiological data associated with four different types of device associated HAI in an ICU at the National Research Center for Oncology and Transplantation (NRCOT) in Astana, Kazakhstan. The information summarized here will form the basis for a much larger surveillance study that could guide decisions regarding appropriate and potentially more efficacious use of prophylactic control within the ICU. This study could also guide the establishment of a national surveillance program to control and prevent HAI in hospitals in Kazakhstan and worldwide.

## Methods

This retrospective, observational study was conducted from January 2014 through January 2016 in a 12 bed ICU at the National Research Center for Oncology and Transplantation (NRCOT), which is a 280-bed hospital in Astana, Kazakhstan. We undertook this pilot study comparing HAI cases and causative agents over a two year period to assess the current local infection control practices. We hope to understand from this study if intervention in the future would be appropriate to improve upon the current HAI scenario. The main change between 2014 and 2015 was access to better diagnostic capabilities within the hospital laboratory, though there are future plans to implement an improved HAI prevention program. This was considered by us to be a “retrospective” study because data analysis on the most part was done after these were collected (even though the staff collected the data during the patients’ ICU visit). A standard screening protocol for HAI was used [13]. We tested all patients who spent more than 48 h in the ICU (whereby, all patients with less than 48 h stay in the ICU were excluded from this study). Samples for microbiological culture and analysis were obtained from patients who presented with HAI symptoms. Blood count, blood biochemistry, and blood coagulation tests were performed on all suspected patients who were at high risk of developing HAI following guidelines provided by the Healthcare Infection Control Practices Advisory Committee [14]. Clinical Pharmacologist and Hospital Infection Specialists visited the ICU every day. Chest radiography, deep tracheal aspirate, bronchoalveolar lavage (BAL) or mini-broncho-alveolar lavage (mini-BAL) were taken for pathogen identification if VAP was suspected. Blood samples, removed intravascular catheters, urine, urinary and wound catheters were also cultured for microbiological analysis if BSI, UTI or SSI were suspected. Samples were cultured using standard microbiological methods; isolated bacteria were identified by standard microbiological methods and tested for antibiotic susceptibility using Kirby-Bauer disk-diffusion technique according to Clinical and Laboratory Standards Institute (CLCI) specifications [15, 16].

### Study ethics

Ethical clearance for this study was obtained from the Ethics committee of the Institutional Review Board of Nazarbayev University. The study was exempt from being classified as human subject research as no personal information related to any of the patients was made available to the Investigators at any time before, during or after the study.

### Study population and microbiological culture and analysis

The patients admitted to the NRCOT ICU were from different surgical departments, which included the department of general, oncology, transplant, vascular, orthopedic, and gynecology surgeries. The patients with a diagnosis of HAI received treatment according to the hospital’s standard protocol of management of HAI. Samples were taken from all patients admitted to the ICU aged 18 to 90 years of age who developed HAI based on clinical, laboratory and instrumental findings throughout the study duration. For microbiological culture and sensitivity testing, lower respiratory tract secretion, blood and urine samples were taken for diagnosis of VAP, catheter-related BSI, and catheter-associated UTI. Blood culture diagnostics was done for all samples from patients with suspected catheter-related blood stream infections. Data relevant to the diagnosis of HAI were taken by the hospital staff, but only microbiological data regarding etiological agents related to device associated HAI were provided for analysis in this study.

### Statistical analysis

The rates of development of each type of HAI (BSI, VAP, UTI and SSI) as well as the total rate per 1000 cases were calculated. Relative risk for these groups was assessed using StataMP 13.0 software, and statistically significant cutoff was determined at a *p*-value of < 0.05, and highly statistically significant cutoff at a p-value of < 0.001. Prevalence of certain isolated strains of bacteria that caused hospital-acquired infections for each year was determined in percent and compared between the years 2014 and 2015 using Chi-square analysis in StataMP 13.0 software. Statistically significant level was determined at a *p*-value < 0.05; and highly statistically significant level at a *p*-value of < 0.001.

## Results

### General overview of ICU patients and the proportions with HAI

A total number of 1257 patients were admitted to the ICU from January 2014 to January 2016 of which, 56.6% (711 patients) were admitted in 2014 compared to the remaining 43.4% (546 patients) in 2015. The mean number of ICU stay was 18 days (with a range of 1–330 days) in 2014, while in 2015 the mean ICU stay was 36 days (with a range of 12–62 days); these variations are also a reflection of some changes in perioperative care that took place in between these two time periods. The change we mention here that led to increased mean number of ICU stay in 2015 compared to 2014 was due to the change in patient population. In 2014, the patient population consisted of patients who were transferred to the ICU after emergency surgeries (which were more than 85% of all transfers to ICU) such as patients who underwent appendectomy, cholecystectomy, hernia repair surgeries, etc. In 2015, the ICU patient population consisted of patients who were transferred to the ICU after oncological surgery (50%) and transplant surgery (30%). The period of ICU stay of patients after emergency surgery was generally shorter than the patients who were moved to the ICU after oncological or transplant surgery. Overall, the patients’ general health conditions were better for the patients in 2014.

During the study period, 249 out of 1257 patients admitted to the ICU developed HAI; the *p*-value for the development of HAI between 2014 and 2015 was 0.000127 which is highly statistically significant. A total of 114 microbiological cultures were obtained and identified from patients suspected to have had HAI in 2014 compared to 135 cultures isolated in 2015 (with a *p*-value of 0.6582 which is not significant).

### Device associated infections

The HAIs were classified into four categories: BSI, VAP, UTI and SSI. As evident from the Table 1, in 2014, SSI constituted the highest percentages of all HAIs followed by VAP, BSI and UTI. When compared to 2014, similar profile of device associated HAIs were observed for 2015, whereby SSI was again the most prominent category that caused HAI, followed by VAP. However in 2015, the percent of observed BSI was equal to the percent of UTI as the least frequent types of HAI.

**Table 1:** Types of Hospital Associated infections in percent for each year

Type of infection	Percent in 2014 (total number)	Percent in 2015 (total number)
BSI	24.6% (28)	18.5% (25)
VAP	25.4% (29)	29.6% (40)
UTI	19.3% (22)	18.5% (25)
SSI	30.7% (35)	33.3% (45)
Total	100% (114)	100% (135)

*BSI*: Catheter-associated Blood Stream Infections, *VAP*: Ventilator-associated Pneumonia, *UTI*: Catheter-associated Urinary Tract Infections, *SSI*: Surgical Site Infections

### Variation in types of infections

**Fig. 1:**
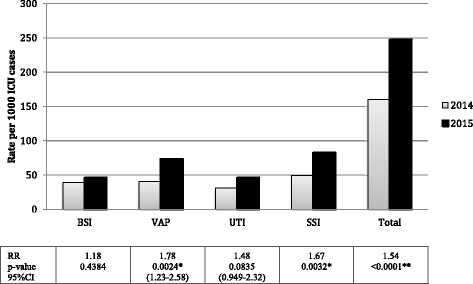
Samples for microbiological culture and analysis were obtained from patients who presented with HAI symptoms. Blood samples, removed intravascular catheters, urine, urinary and wound catheters were also cultured for microbiological analysis if BSI, UTI or SSI were suspected. Comparison of rates of case-specific infections between 2014 and 2015 were performed. Statistically significant level was determined at a *p*-value < 0.05; and highly statistically significant level at a *p*-value of < 0.001. Statistically significant difference is noted for VAP, SSI and combined total between years 2014 and 2015 (statistical significance: *p-value< 0.05; **p-value< 0.001)

Analysis of HAI categories (BSI, VAP, UTI, and SSI) in 2014 compared to 2015 was conducted and revealed significant variation between certain types of HAI (Fig. 1). Specifically, rates of VAP and SSI were found to be statistically different (*p*-value < 0.05) between the two years of the study period. Rates of VAP infections were higher in 2015 than in 2014. Similar results were obtained for SSI, whereby the rate of infection was again higher in 2015 (*p*-value < 0.05). On the other hand, no difference in rates of BSI and UTI was observed. The total rate of all types of infections was found to be significantly higher in 2015 when compared to the total infection rate in 2014 (*p*-value < 0.05). Data was available for the VAP incidence which was determined to be 8.4 per 1000 ventilator days while the incidence of catheter-related blood stream infections was 18 per 1000 line days.

### Microbiological etiology of HAI

**Fig. 2:**
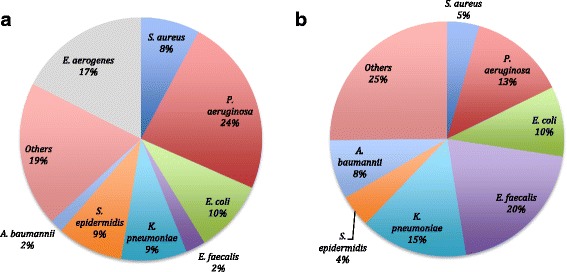
Samples for bacterial culture and analysis were obtained from patients who presented with HAI symptoms. Samples were cultured using standard microbiological methods; isolated bacteria were identified by standard microbiological methods according to Clinical and Laboratory Standards Institute (CLCI) specifications [12, 13]. For comparison, Chi-square test was conducted with a *p*-value < 0.001**. The subgroup ‘Others’ includes rare strains of bacteria (with incidence of < 5%), which were different for each year (comparing 2014 and 2015).

**Fig. 3:**
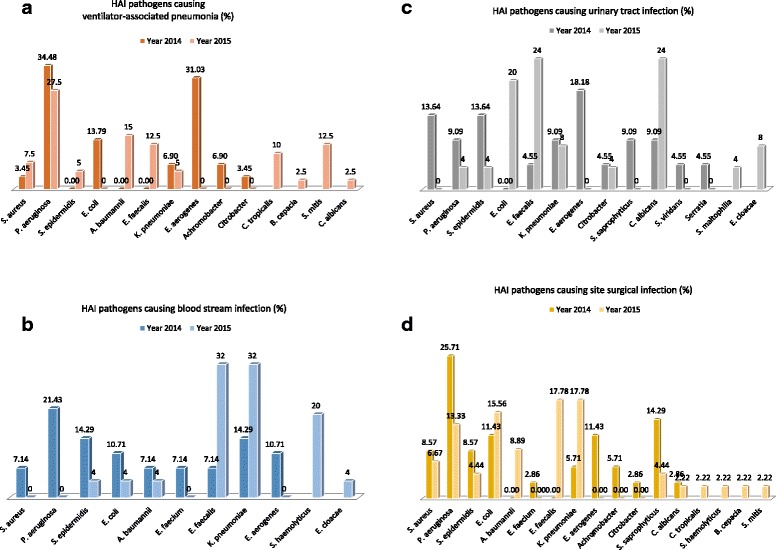
The types of HAI pathogens shown in Fig. 2 were subdivided into the types of HAI samples they were isolated from; **a** VAP, **b** BSI, **c** UTI, and **d** SSI and represented as percent (%) of total number of bacteria isolated. Samples were cultured using standard microbiological methods; isolated bacteria were identified by standard microbiological methods according to Clinical and Laboratory Standards Institute (CLCI) specifications [12, 13]

A total number of 249 microbial pathogens were isolated and identified in 2014 and 2015, and the percent distribution of these etiological agents is represented in Fig. 2a and b. The highest percent in 2014 corresponded to *Pseudomonas aeruginosa* at 23.68%, which was slightly reduced in 2015 to 13.33%. *Klebsiella pneumoniae*, *Escherichia coli*, *Staphylococcus aureus*, *Staphylococcus epidermidis,* and *Acinetobacter baumannii* were isolated and identified from many HAI cases for both 2014 and 2015. Unique and relatively widespread isolate for 2014 was *Enterobacter aerogenes*, representing 17.54% of the cases. Bacterial strains that had an incidence of less than 5% were combined into the subgroup ‘Others’. In 2014 *Streptococcus viridans, Citrobacter* spp., *Enterococcus faecium, Achromobacter* spp., *Staphylococcus saprophyticus, Candida albicans and Serratia* spp. were included in this subgroup. In 2015 however, in addition to the above-mentioned *Citrobacter* spp.*, S. saprophyticus* and *C. albicans,* unique pathogens such as *Staphylococcus haemolyticus*, *Enterococcus cloacae*, *Candida tropicalis*, *Burkholderia cepacia*, *Streptococcus mitis* and *Stenotrophomonas maltophilia* were isolated. When comparison analysis was conducted, highly significant difference between two years (*p*-value < 0.001) was identified. Even though many strains were similar for both years, case-wise analysis revealed important differences that should be taken into account. The types of pathogen described originated from cultures isolated from the different HAI categories as shown in Fig. 3a (VAP), Fig.3b (BSI), Fig. 3c (UTI), and Fig. 3d (SSI), and represented as percent (%) of total number of organisms isolated. We found that HAI were predominantly caused by gram-negative bacterial pathogens, particularly *P. aeruginosa* and *K. pneumoniae*. These strains were most frequently associated with all the HAI types particularly SSI and BSI (as the first two most common types of HAI pathogens isolated) though there are some variations between the two years with these two pathogens as well as other types of bacterial pathogens that predominated, such as *Staphylococcus epidermidis, E. faecalis* and *Acinetobacter baumannii*. From our study, *P. aeruginosa* was recognized as a causal agent of the most serious HAI in the ICU. This organism was also causing the majority of VAP and BSIs in two consecutive years. SSI analogously was mainly caused by gram-negative bacteria, of which *P. aeruginosa* prevailed in 2014, while *K. pneumoniae* predominated in 2015.

## Discussion

In this study, we have shown that healthcare-associated infections constitute a major healthcare burden in Kazakhstan, which is a rapidly developing nation. Kazakhstan is one of the five Central Asian countries (which also include Kyrgyzstan, Tajikistan, Turkmenistan and Uzbekistan) that achieved independence in 1991 after the collapse of the Soviet Union [17, 18]^.^ At independence, these Central Asian Post-Soviet (CAPS) countries were faced with many problems; progress to build stronger economies varies between countries. Collapse of the Soviet Union led Kazakhstan as well as the other CAPS countries to economic recession. During the Soviet Union era, there were no wide informational, medical and pharmaceutical exchanges with other countries. On the other hand, the dissolution of the Soviet Union mobilized these countries to democracy which initiated important exchanges of products and knowledge with the other countries. Greater successes have been noted for Kazakhstan, Turkmenistan and Uzbekistan, which are the CAPS countries with the richest natural resources [17]. The healthcare systems of these CAPS countries have also gone through many decades of profound revolutions, whereby the rate and quality of progress of the healthcare sectors vary between these countries [19]. Since independence, the CAPS countries have steadily been receiving more attention at the international arena, especially due to the economic and geographical significance of the region. Over the last two decades, Kazakhstan has made significant progress in economy as well as healthcare sector, although there is still much room for improvement.

To our knowledge, this is the only study to be reported in an international journal describing ICU-related HAI situation from the Central Asian region. In our study, HAI represents a significant healthcare problem with ICU related HAI incidence being about 2 to 5 times higher than the incidence in other in-hospital departments [7]. In our study, patients frequently had the following universal host-related risk factors predisposing to HAI which included advanced age, severity of disease, hyperglycemia, neutropenia, immunosuppressive therapy, cancer diagnosis, and malnutrition. Majority of the patients had at least three of these risk factors. The most common type of HAI in our study was SSI whereby the rate increased significantly (*p*-value < 0.05) when comparing the year 2014 to 2015 (Table 1 and Fig. 1). Between both years, no intervention was introduced in terms of recommendations to improve upon infection control practices which may have been one of the reason there were no observed decrease in device-associated HAI rates. This high incidence of HAI associated with SSI was due to the fact that the Astana NRCOT is a multidisciplinary hospital focusing on multiple types of surgeries. In most cases, patients also had poor nutritional and altered immune status due to devastating diseases, which were either cancer, kidney and liver failure, diabetes, and coexisting infections at the remote body site. Multiple studies stated that the above mentioned medical conditions predispose patients to the development of SSI [20, 21]. Medical procedures in the hospital were typically prolonged, and patients were prone to intraoperative hypothermia, increasing the risk for SSI [22]. In our setting, colorectal surgery was the most frequently performed surgery, which is recognized as a risk factor for SSI worldwide [23].

From our study, VAP was identified as the second most common type of HAI. For patients on mechanical ventilation however, VAP was the leading type of infection, which is consistent with reports from multiple other studies [24, 25]. Majority of the patients who developed VAP had COPD, advanced age, H_2_-antagonists and antibiotics use, and Multiple Organ System Failure [26]. Similar to the SSI associated HAI described above, the rates of VAP also increased significantly when comparing 2014 to 2015 (Table 1 and Fig. 1); this may have also been due to absence of intervention that was implemented to reduce the number of VAP cases in the studied ICU. The third most common type of HAI identified in our study was BSI. Almost all patients who developed BSI had the following risk factors; central vein catheterization, malnutrition and surgery, and infection with highly antibiotic resistant pathogens. These findings are supported by evidence from other studies, which report BSI to occur in 5% of all ICU patients [26, 27].

We found that HAI were predominantly caused by gram-negative pathogens, including *P. aeruginosa*, *K. pneumoniae*, and *E. coli.* These strains were most frequently associated with VAP, SSI, BSI and UTI. From our study, *P. aeruginosa* was recognized as a causal agent of the most serious HAI in the ICU [28]. This organism was also causing the majority of VAP and BSI in two consecutive years [9]. SSI analogously was mainly caused by gram-negative bacteria, of which *P. aeruginosa* prevailed in 2014, while *K. pneumoniae* predominated in 2015. Another interesting finding was with regards to the differences between the length of ICU patient stay, the patient population type, rate of HAI as well as the types of pathogens isolated in 2014 when compared to 2015. Since the period of ICU stay of patients after emergency surgeries was much shorter than after oncological or transplant surgeries, the overall period of ICU stay was longer in 2015. Furthermore, ICU patients’ general condition was better among patients in 2014. Our results showed that the rate of HAIs was increased and the distribution of pathogen also changed in 2015 compared to 2014. We propose that the difference between patient populations in the ICU partially explained the reason for increased number and rates of HAI in 2015 (Table 1 and Fig. 1), as well as the differences in the types of HAI pathogens that were isolated when these pathogen types were compared between the two years of study. With regards to increased HAI rate, both the oncological and transplant patient populations were immunocompromised due to the surgical procedures, and these patients also received immunosuppressant drugs; this led to further decrease in their immune status. Plus, these patients stayed longer in the ICU thereby had higher risk of acquiring ICU-related HAIs. Interestingly, more HAI pathogens that are more commonly associated with multidrug resistance belonging to the “ESKAPE” pathogen group were identified in 2015. This included *P. aeruginosa, E. faecalis, K. pneumoniae and A. baumannii* (Fig. 2); this is not a surprising finding given that these pathogens are associated with many HAI infections within the last few decades [29].

There are limitations to this study, which include the fact that data collected did not include patients’ age, sex, and other demographic variables, which might have contributed towards generation of more precise and valuable conclusions. For future studies, the design could include collection of these and other relevant information that would enhance the quality of data and conclusions that can be derived from the study. In this study, some organisms identified were usually not considered common causative agents (such as *Candida*, *Enterococcus faecalis, Staphylococcus epidermidis* and *Streptococcus mitis* for VAP as in Fig. 3a, and *S. epidermidis* and *S. viridans* for UTI as shown in Fig. 3c). Such unexpected findings further support our conclusion that there is an urgent and definitive need to improve upon HAI prevention practices which include incorporation of surveillance protocols that meet international standards that can more accurately detect and identify the correct etiological agent (which includes incorporation of quality control measures and established Standard operating procedure) in Kazakhstan and other developing countries within the region. These findings indicate that the surveillance protocols mentioned in the method section were not fully implemented during the study period, which is a major limitation of this study. Therefore the findings from this study demonstrates the need to improve training of infection control personnel regarding the use of and implementation of more stringent practices that meet the international HAI infection control and surveillance guidelines.

HAI is a critical problem for health-care providers worldwide, which should receive appropriate attention for further management. The integrated HAI control program was introduced more than 3 decades ago for the first time, being able to reduce both incidence of infections and related healthcare costs [30]. Unfortunately, many developing countries such as Kazakhstan lack surveillance systems which could effectively decrease incidence of HAIs and healthcare costs for their treatment. Currently, the international guidelines on prevention, diagnosis and treatment of HAI are not actively practiced, and access to newer antibacterial agents is not readily available in Kazakhstan. Within Kazakhstan, there are National and Regional Medical Centers (hospitals). There are no published epidemiological data on HAI from the Regional Medical Centers, therefore, the epidemiological characteristics of HAI from these Centers are unknown. If we compare the National Medical Centers with Regional hospitals, there is an unequal supply of medical equipment and uneven qualification of medical doctors and staff in the latter. For example, several National Medical Centers have internationally educated doctors and staff as well as equipment resembling those that can be found in the best World Class Medical Centers. These medical Centers can be effective in monitoring, diagnosing, treating and preventing the HAI, whereas many Regional (and rural) hospitals do not even follow any guidelines on diagnosis, treatment and preventions of HAIs. The National Medical Centers frequently receive severely ill patients with multiple undiagnosed multi- and pandrug resistant HAI from the Regional Medical Centers.

We propose that the most important recommended measures to prevent HAI at the NRCOT and similar Healthcare Institutions are rational antibacterial therapy, regular infection control audits, and general hygiene measures. Numerous studies in developing countries have shown that the International Nosocomial Infection Control Consortium (INICC) multidimensional infection control strategy with practice bundles can decrease the rate of HAI. Rosenthal et al. found that implementation of a multidimensional infection control strategy can significantly reduce the central line-associated BSI rates in the PICUs of developing countries [31]. In another study, Tao et al. demonstrated that a multidimensional infection control intervention for VAP contributed to a significant cumulative reduction in the VAP rate in their ICUs [32].

This is a first surveillance study on HAI at the NRCOT since its foundation eight years ago. An active surveillance system monitoring incidence and prevalence of HAI as preventative measures is necessary, and this study represents a first step towards this goal.

## Conclusions

We conclude that HAI is one of the major problems in healthcare provision in Kazakhstan. SSI, VAP and BSI are the predominant types of HAI. Gram-negative bacteria such as *P. aeruginosa*, *K. pneumoniae* and *E. coli* are the most common causative agents of HAI. The most important risk factors are advanced age, severity of disease, hyperglycemia, neutropenia, immunosuppressive therapy, cancer diagnosis, and malnutrition. For more precise determination of its causality, an ongoing HAI surveillance program is needed to decrease HAI incidence and prevalence at the NRCOT and other healthcare institutions throughout the country. Kazakhstan has made significant effort and progress since independence to establish a healthcare system that is already an improvement over the centralized Semashko health system model inherited from the Soviet Union at independence [19]. One of the ways to continue on the positive trajectory of change for an improved healthcare system is implementation of an efficient active surveillance system for HAI that should be implemented nationwide. The system should include an organized and controlled data collection performed by trained personnel. The data collected should include not only infection-related data, but also patient-related data such as risk factors and history of present illness (such as history of HAI, diagnostic tests, surgeries and invasive methods, as well as the antimicrobial drugs used). A system of monthly HAI analysis and report should also be implemented. Such proactive surveillance system coupled with efficient infection control programs would be highly beneficial to control and reduce the rates of HAI within all Regional and National Medical Centers throughout Kazakhstan.
